# Depressive symptoms of school-aged adolescents: cumulative effect of adverse events and mediation of social support

**DOI:** 10.1590/1980-549720250019

**Published:** 2025-05-09

**Authors:** Célia Regina de Andrade, Raquel de Vasconcellos Carvalhaes de Oliveira, Joviana Quintes Avanci

**Affiliations:** I Fundação Oswaldo Cruz, Sergio Arouca National School of Public Health, Graduate Program in Public Health, Department of Quantitative Methods in Health – Rio de Janeiro (RJ), Brazil.; II Fundação Oswaldo Cruz, Evandro Chagas National Institute of Infectious Diseases. Graduate Program in Public Health Epidemiology – Rio de Janeiro (RJ), Brazil.; III Fundação Oswaldo Cruz, Sergio Arouca National School of Public Health, Graduate Program in Public Health, Jorge Careli Department of Violence and Health Studies – Rio de Janeiro (RJ), Brazil.

**Keywords:** Adolescence, Depression, Social support, Adverse childhood experiences

## Abstract

**Objective:**

To analyze the effect of cumulative adverse childhood experiences and depressive symptoms on adolescence and to identify direct and indirect relationships between the variables and the mediating role of social support.

**Methods:**

This is a cross-sectional study with a sample of 1,117 school-aged adolescents aged 13 to 19 years. The Children's Depression Inventory and Adverse Childhood Experiences scales were used, organized into socioeconomic, family, community, and total dimensions. Descriptive analysis was carried out and the structural equation model was used, with probit estimation, applying social support as mediator.

**Results:**

The prevalence of depressive symptoms among school-aged adolescents was 8.2%. We observed that the accumulation of socioeconomic, family, and community factors increases the occurrence of depressive symptoms, adjusted for sex, skin color, and social support. Mediation by social support explains 16.7% of the total effect of exposure to the analyzed factors. In the socioeconomic dimension, the depression probit increases by 0.033 for each category, with 21.2% mediated by social support. In the family dimension, the increase is 0.020 (25% mediated); in the community, 0.018 (16.7% mediated); and in the total dimension, 0.012 (17% mediated).

**Conclusion:**

The observation of the effect of the accumulation of Adverse Childhood Experiences on depressive symptoms in adolescence requires actions in vulnerable groups, with social support helping to mitigate the risk of depression.

## INTRODUCTION

Depression has become relatively common throughout the world, and in the last decade it has increased by almost 20%, with an estimated 322 million individuals or 4.4% of the world's population with depressive symptoms^1^. Brazil ranks second among the largest number of people with depression in the Americas, ranking first in the comparison between Latin American countries^1^.

Throughout the world, adolescents are deemed a risk group for depression, with an estimated 6.2 to 25% for major depressive disorder^2^. The cumulative risk of occurrence of one or more depressive episodes increases from five to 20% at this stage of life^3^. In adolescence, depression is associated with school and social difficulties, poor physical health, suicide, and recurrence of depressive episodes in adult life^2,4^. The risk factors that stand out are the family history of depression and exposure to Adverse Childhood Experiences (ACEs)^5^.

ACEs refer to trauma or negative experiences that occurred up to 18 years of age, with the potential to harm health and well-being in the long term^6^. Unemployment, violence, diseases, losses, dysfunctional family environments, and family structure instability are studied adversities that can accentuate depression in adolescents^7^. In addition, community violence, racial discrimination, and poverty are also considered ACEs^7^.

In Brazil, the situation of children, adolescents, and their families has worsened in recent years. Sexual exploitation, abandonment, losses, hunger, and violence significantly affect this group. Between 2016 and 2020, 35 thousand young people aged 0 to 19 years were killed violently. In addition, from 2017 to 2020, 180 thousand young individuals suffered sexual violence — an annual average of 45 thousand per year^8^. The increase in unemployment since 2015, especially among women (16.8%), impacts the lives and health of these young people^8^.

The cumulative ecological risk perspective investigates risk sources from different life contexts, such as family, school, community, and peers, which operate simultaneously or in sequence^9^. This theory, based on stress response, suggests that cumulative exposures affect physiological regulatory systems in the long term^10^. This may provide for persistent psychopathology from childhood to adulthood^11^ and psychiatric disorders in adolescence^12^. Stress generated by ACEs provides for 21% of depression symptoms in adolescents^13,14^.

However, little is still known about the mediating and moderating mechanisms underlying the relationship between depression and cumulative risk of ACEs, which could indicate potential protective factors for preventing depression. These factors can modify or improve the response to an adverse event and prevent the development of depressive symptoms^15^.

The ecological-transactional model of the developmental psychopathology organizes protection factors at various levels:

1. The individual, such as the skills of positive coping, self-regulation, internal locus of control, spirituality, and high self-esteem;
2. The familiar, among which support relationships; and
3. The wider community-related, such as the connection with cultural activities or groups, the high social cohesion of a neighborhood, and community support^15^.

Social support is a protective factor studied in theoretical models on depression. The social causality model suggests that social support, by providing positive experiences of affection and care, attenuates the probability of depression, strengthening self-esteem and reducing negative cognition^16^. Reduced levels of perceived support provide for depressive symptoms in adolescence^17^. In addition, adolescents who realize that they can rely on trustworthy people are able to reduce the negative effects of depression caused by stressful life events^17^.

It is strategic to understand the role of potential adversities in mental health outcomes as well as to reflect on the effect of the accumulation of adverse experiences and the occurrence of depression in adolescents^18^. It is even more strategic to know the mediating role of protective factors, such as social support in the face of exposure to adverse situations, seeking to guide intervention actions aimed at children and adolescents exposed to different forms of ACEs^19^. In this article, we sought to analyze the effect of cumulative adverse childhood experiences and depressive symptoms on adolescence and to identify direct and indirect relationships between the variables and the mediating role of social support.

## METHODS

### Study design and population

This is a cross-sectional study on adolescents from public and private schools in the city of São Gonçalo (State of Rio de Janeiro – RJ) conducted in 2011. This study is part of a larger project developed by the Jorge Careli Department of Violence and Health Studies of Fundação Oswaldo Cruz (Fiocruz).

The municipality of São Gonçalo is the second most populous in the State of Rio de Janeiro^20^. At the time of the research, it had an estimated population of 999,728 inhabitants^20^, of which one third are children and adolescents (328,829) aged from 0 to 19 years, and population in the last census (2022) of 896,744 inhabitants^20^. The Human Development Index (HDI) was 0.739 in 2010, occupying the 14th place among the cities of Rio de Janeiro. The adolescence age group represents about 16% of the population of the municipality and, in 2022, the infant mortality rate was 12.56 per one thousand live births^20^. Regarding violence against children and adolescents, in 2017, the *Disque 100* [Dial 100], the channel of the National Ombudsman for Human Rights, recorded more than 84 thousand complaints of violation of the rights of children and adolescents, with more than 20% related to sexual violence^21^.

Despite the high rates of violence against children in the city, public policies aimed at children and adolescents who are victims are still incipient. It is worth mentioning the Special Center for Assistance to Children and Adolescents Victims of Violence (*Núcleo Especial de Atendimento a Crianças e Adolescentes Vítimas de Violência* – NEACA), part of the intersectoral flowchart, and the Sentinel Program (*Programa Sentinela*), which contribute to the network of care and protection to these young people^20^.

The sample included adolescents from 13 to 19 years old, students from the 9th grade of public and private schools in the city in 2011. Composed of 694 students from public schools and 423 from private schools, the sample was sized to estimate a 2.3% proportion, with a 1.3% error, and a 95% confidence level. A total of 14 strata were considered according to the planning areas and the type of school. A multistage cluster sampling was used, with random selection of schools and classes. In total, 1,117 adolescents from 43 public schools and 30 private schools participated in the research, with about two classes per school.

The research was approved by the Ethics Committee of the National School of Public Health/Fundação Oswaldo Cruz (Fiocruz). The management of the schools and the guardians signed the Consent Form, and the adolescents, the Assent Form.

### Research instruments

The self-administered questionnaire was applied per class, by trained teams, being one of the members a psychologist. The questionnaire was composed of items and scales previously validated and used in international studies and with the Brazilian population, in which the considered exposure variable was the ACEs.

#### Adverse Childhood Experiences (ACEs)

A total of 33 items were investigated based on two dimensions: traumatic life events^22^ — going through a landslip, disaster, fires, floods; serious accident; armed conflict or shooting; the teenagers themselves/a family member being beaten, punched, or kicked at home; being beaten, shot, or threatened; witnessing someone being beaten, shot, or killed; seeing a corpse in your town; having your intimate sexual parts touched by an adult; having news of a violent death or serious injury of a loved one; having received frightening and painful medical treatment in a hospital when you were very ill or seriously injured; having experienced another frightening, dangerous, or violent situation; and the scale by Trombeta and Guzzo^23^, with the following items: unemployment of one of the parents/guardians; serious financial problems in the family; lack of food at home; lives or has already lived crowded, without space; serious medical problems of family members; physical or mental problem in the family; someone in the family is indicted or under arrest; death of the father or mother; death of a close relative; alcohol or drug problem in the family; sexual experience that involved the parents; discrimination based on skin color; separation from friends due to a fight or death; seeing someone seriously injured; living in danger or insecurity in the neighborhood; having one's house robbed or burgled.

#### Severe violence of the mother and/or father against the adolescent

The Tactical Conflict Scale^24^ was used, validated in Brazil^25^, composed of six items ranging from mild to severe physical aggressions. The answers range from "often" to "never," and a positive response indicates the presence of violence. In a similar study, the Cronbach's alpha found for severe violence was 0.85 for mothers and 0.92 for fathers^26^.

#### Psychological violence^27,28^

It is characterized by 18 items related to acts of humiliation, excessive criticism, and the use of derogatory words against the adolescent committed by significant people, with options of answers that vary from never to always. It was tested as for construct validity, with positive correlation with psychic suffering^29^, violence committed by the mother, and between parents^25^. Kappa ranged from 0.395 to 0.683 and the verified Cronbach's alpha was 0.930^28,29^.

#### Violence in school and in the locality

The Scale of Violence in School and in the Community of the United Nations Latin American Institute for the Prevention of Crime and the Treatment of Offenders (ILANUD)/United Nations (UN)^30^ assesses the victimization of adolescents in school and community in the previous year. A positive item characterizes the adolescent as a victim of violence in the corresponding context. The Kuder-Richardson coefficient was 0.52 for school violence and 0.57 for community violence, acceptable due to the low number of items^30^.

#### Violence between parents (or stepfather/stepmother) and between siblings

It was evaluated by fights to the point of hurting and humiliating themselves. One or more positive responses in each relationship makes it a case in the measured victimization^26^. Good psychometric coefficients were obtained in the evaluation of these constructs: in the violence between siblings, the obtained Cronbach's alpha was 0.83, the intraclass correlation index (ICC) was 0.6, and moderate kappa; as for the violence between parents, the ICC was 0.68^30,31^.

The 28 items of ACEs and violence were organized and evaluated by:

1. Total score, measured by the sum of all positive items; and
2. Three-dimensional scores — socioeconomic, family, and community (Table 1).

**Table 1:** Prevalence of Adverse Childhood Experiences according to depressive symptoms among 1,117 school-aged adolescents in São Gonçalo, Rio de Janeiro, Brazil.

Adverse experiences		Symptoms Presence Prev.% (n=92)	Depressive Absence Prev.% (n=1025)	p-value*
Socioeconomic dimension				
	Your parent or guardian is unemployed	67.2	58.0	**0.061**
	Serious financial problems	47.4	37.1	**0.057**
	Lack of food at home	5.6	2.1	**0.081**
	Lives or have lived in a crowded place	12.5	6.7	**0.071**
Family dimension				
	Have been beaten, punched or kicked hard at home	37.3	18.3	**<0.001**
	An adult has touched your intimate sexual parts against your will	17.0	7.7	**0.008**
	Family member indicted or under arrest	34.6	20.7	**0.007**
	Have seen a family member being beaten, punched or kicked hard at home	39.6	22.6	**<0.001**
	Psychological violence	55.3	14.4	**<0.001**
	Have received news of violent death from a loved one	38.9	41.7	0.656
	Medical problems of family members	66.7	69.5	0.586
	Physical or mental disability of a family member	33.5	24.0	**0.071**
	Death of the father or mother	6.5	6.8	**0.928**
	Death of a close relative	75.1	72.4	0.625
	Alcohol or drug problems of family members	41.6	31.2	**0.059**
Community dimension				
	Have been beaten, shot or threatened	30.2	10.8	**<0.001**
	Violence at school	61.9	38.6	**<0.001**
	Violence in the locality	64.6	35.9	**<0.001**
	Have been separated from friends due to a fight or death	62.8	30.9	**<0.001**
	Have seen someone being seriously injured	48.2	37.0	**0.045**
	Situation of danger and insecurity in the neighborhood	33.1	25.5	0.129
	The house has been robbed or burgled	12.3	11.6	0.825
	Disasters/fires/floods	21.0	12.1	**0.049**
	Have been in a serious accident	19.3	11.3	**0.015**
	Have been in armed conflict (shootings)	39.6	35.9	0.484
	Have seen someone in your city being beaten, shot, or be killed	47.2	34.2	**0.053**
	Have seen a corpse in your city	56.7	49.9	0.390
	Landslide that destroyed the place you have been	10.8	5.4	**0.058**

Prev.: prevalence;

* values in bold represent p≤0.10, statistically significant tests.

The options of ordinal responses of the socioeconomic dimension were 0–2 and more; of the family dimension, from 0 to 2–5 and more; of the community dimension, from 0 to 2–5 and more; and of the total dimension, from 0 to 7–8 and more (Table 2).

**Table 2:** Modeling of the effect (total, direct, and indirect) of the score of the dimensions of Adverse Childhood Experiences on depressive symptoms mediated by social support between school-aged adolescents (n=1,117).

Dimension		Total effect (c’+ab)	Direct effect (c’)	Indirect effect (a*b)	Mediation proportion†%
Socioeconomic‡					
	0	–	0.026	0.007	21.2
	1	0.033			
	2 and more	0.066			
Family§					
	0 to 2	–	0.015	0.005	25.0
	3 to 4	0.020			
	5 and more	0.040			
Community //					
	0 to 2	–	0.016	0.003	16.7
	3 to 4	0.018			
	5 and more	0.036			
Total^g^					
	0 to 7	–	0.010	0.002	16.7
	8 and more	0.012			

* The effects were adjusted by sex and self-reported race/skin color;

† Mediation Proportion=indirect effect (a*b)/total effect (c’+ab);

‡ Socioeconomic Model CFI=0.972, RMSEA=0.048;

§ Family Model CFI=0.971, RMSEA=0.058;

// Community Model CFI=0.987, RMSEA=0.037;

¶ Total Model CFI=0.986, RMSEA=0.043.

#### Depressive symptoms

The Children's Depression Inventory (CDI), developed by Kovacs^32^, evaluates the presence and severity of depressive symptoms in children and adolescents, adapted from the Beck Depression Inventory (BDI) for adults. In Brazil, it was translated and adapted by Gouveia et al.^33^, keeping the 27 items from the original version. The CDI allows the screening of depressive symptoms in individuals aged 7 to 17 years, addressing affective, cognitive, somatic, and behavioral symptoms^34,35^ in the last two weeks. The response scale has three options: absence, presence, and severity^35^. Its psychometric properties are well established, with alpha coefficients ranging from 0.18 to 0.55^36,37^. The score is obtained from the sum of the items, with values above 65 indicating possible cases of depression^37^.

#### Social support

The reduced version of the social support subscale of the Medical Outcome Study Social Support Survey (MOSSSS), with six items and one factor^38^, was adapted and validated for Brazil^39^, presenting good reliability and validity measures^40,41^. This version evaluates the global functional social support (material support, affective support, positive social interaction, and emotional/informational support) and shows high internal consistency. The average is used as a cutoff point to classify social support into two categories: from 7 to 25 points, absence of social support; from 25 to 30, presence of social support^39^.

The variables sex (boys and girls) and self-reported skin color (white, Black/mixed-race, and Asian/Indigenous) were studied as sociodemographic covariates.

### Data analysis

The questionnaire items were coded and typed into a data masking in Epidata 3.1. Descriptive analysis was performed to obtain the prevalence of depressive symptoms and other variables. Subsequently, the items of ACEs and types of violence were associated with the variable of depressive symptoms (present/absent), comparing the proportions by the Wald test, with a 10% significance level.

Significant variables at the level of 10% were selected to create the four exposure variables related to ACEs: three dimensions (socioeconomic, family, and community) and the total score. The eight ACEs events (such as death of loved ones, medical problems of family members, and armed conflicts) were included in the dimensions for their theoretical relevance. Each exposure variable was created by adding the events (one if present, zero if absent) and then categorized into two or three categories according to the number of events.

Subsequently, the model of structural equations for binary outcome (with probit binding function) was applied to evaluate the effects of exposure of each of the three dimensions and the total score of ACEs on the outcome of depressive symptoms. Exposure variables (ACEs) were treated as ordinal variables in the estimation (Table 2).

**Figure 1:**
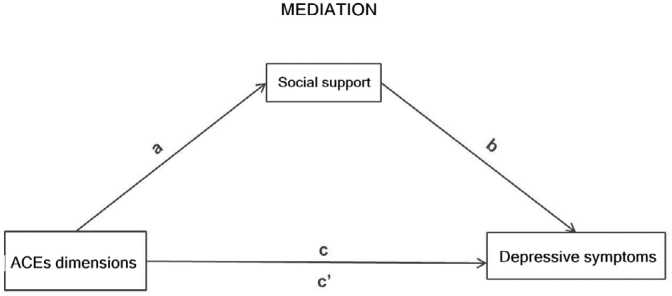
Diagram of the effect (total, direct, and indirect) of the score of the dimensions of Adverse Childhood Experiences on depressive symptoms mediated by social support.

Note: a=exposure effect - >mediator; b=mediator effect; c=total effect; c’=direct effect or average direct effects (ADE)

The effects were interpreted with each change in the category of ACEs dimensions and the models were adjusted by the variables sex (boys and girls) and self-reported race/skin color (white and Black — Black/mixed-race/Asian/Indigenous), besides considering the variable social support as a measuring variable (Figure 1). The 5% significance level was considered in the tests of the model coefficients.

a*b=indirect effect or average causal mediation effects (ACME), c=c’ + a*b=average direct effects (ADE)*average causal mediation effects (ACME).

ACEs: Adverse Childhood Experiences.

The total effect of each dimension and total ACEs on the outcome of depressive symptoms was composed of a direct and an indirect effect, that is, mediated by social support (Figure 1). The objective of the mediation analysis was to estimate a partition of the total effect in an indirect effect of exposure on the outcome via mediation and a direct effect of exposure on the outcome^42,43^. If there is an effect of ACEs via mediation (indirect effect), the proportion between the indirect and total effect will be reasonable. Values above 10% of mediation proportion were considered as a potential effect.

To evaluate the model fit quality, the comparative fit index (CFI) and the root mean square error of approximation (RMSEA) measurements were used, considering an adequate fit when CFI was above 0.9 and RMSEA, below 0.05^44^.

The analyses were performed using the survey library^45^ of the R statistical package and by incorporating the sample weight and plan in all of them. For structural modeling, the *lavaan.survey* ^46^ package (version 06-19) of the R software, version 4.4.1 (http://www.r-project.org)^45^, was used.

## RESULTS

Of the 1,117 participants, the prevalence of depressive symptoms was 8.2% (95% confidence interval – 95%CI, 6.7–9.9). Of the 33 adverse events and types of violence studied, we found significant statistical differences in the presence or absence of symptoms of depression in 20 of them. In the socioeconomic dimension, parents’ unemployment, serious financial problems, lack of food, and crowded living stood out. In the family dimension, we highlight victimization or witnessing physical violence, sexual violence, psychological violence, arrest of a family member, physical or mental disability, and problems with alcohol or drugs. In the community dimension, we highlight violence of the community, the school, the separation from friends due to a fight or death, witnessing violence in the city, accident, disasters/fires/floods, and landslide (Table 1).

The ACEs dimensions were created by the sum of events of the 20 significant items in Table 1, in addition to eight items selected by theoretical relevance and, later, categorized. In Table 2, we show the results of the structural equation model applied to each dimension and to the total ACEs score, adjusted by self-reported race/skin color and sex, considering mediation by social support. We observed that the analysis of ACEs has a gradient effect, as it was cumulative according to the categories of each dimension. In the socioeconomic dimension, with each increase of a category, the probit of depression occurrence increases by 0.033, when adjusted by sex and skin color, being, however, 21.2% of this total effect mediated by social support. Regarding the family dimension, the increase is 0.020, and 25.0% is mediated by social support; in the community dimension, the increase is 0.018, and 16.7% is mediated by social support. Finally, in the total dimension, the increase is 0.012, and 16.7% is mediated by social support. The fit quality measurements of the four models were in accordance with the expected parameters (Table 2).

## DISCUSSION

In this study, we bring a relevant contribution to the understanding of the complex relationship between adversities and depression. First, the prevalence of 8.2% of depressive symptoms among school-aged adolescents corroborates mental health surveys in the age group, in which percentages between 7 and 10% were found^47,48^.

However, two main findings stand out for the outcome of depression in adolescence:

1. The gradient of effect due to the accumulation of adverse events; and
2. The mediating role of social support, which attenuates the occurrence of depression, even if it does not prevent its occurrence.

The dose-response relationship between the increase of Adverse Childhood Experiences and the higher occurrence of depressive symptoms in adolescents highlights the cumulative negative impact of these experiences^49^. Each new adverse event in each dimension studied progressively increases the occurrence of depressive symptoms. This finding is relevant, as it points to adolescents who, due to their living conditions or mental health, live in contexts that feed back on adverse experiences, and may trigger/aggravate their current or future condition, including eating and personality disorders^50-52^. The situation is worrisome, because many of these adverse experiences are kept secret and are not recognized or valued by people around the teenagers^13^.

As for social support, its protective role for the development of depression and other mental disorders is known^50^, tending to mitigate the damaging impacts of adversities^18,51^. Teenagers who receive help, support, and are heard can see difficult situations as less threatening and develop more positive attitudes. However, we showed that social support alone does not prevent the cumulative impact of ACEs on the development of depressive symptoms. Experiences of poverty, violence, accidents, losses, and diseases in childhood put adolescents at risk of mental health problems, influencing the subsequent stages of life^52^. The mediating relationship of social support does not depend on the sex or race of the adolescent, which requires caution and further future studies.

In the results found among the dimensions, the greatest effects on depression concerned the socioeconomic dimension, followed by the family dimension. Social support also has a greater mediating effect on the two aforementioned dimensions.

The findings should be studied based on the Dimensional Model of Adversity and Psychopathology^53^, which differentiates threat events (such as physical and sexual violence) and deprivation (such as negligence). This model suggests that threats result in regulation of fear stimuli, while the experience of deprivation impairs cognitive maturation and learning. Different adversities may explain the interactive response of ACEs in depression^54^. Violence is linked to threat processing and depression, to deficits associated with loss^55^, demonstrating that the effects of adversity can be independent and better understood by their nature of adversity^56^.

The main limitation of this study refers to the use of a self-report questionnaire. This type of approach restricts information about the context of events and depression, and is subject to memory biases. In addition, the current mood of the adolescent can lead to excesses of reports of adversity and suffering. It is worth noting that the analysis of mental health issues prior to the COVID-19 pandemic may present a more positive scenario of phenomena such as depression, requiring a more critical understanding based on the current context. As a recommendation for future studies, we suggest the use of longitudinal studies and the application of a qualitative approach, which can refine the understanding of causality of family and social factors that trigger depression in adolescence.

In conclusion, the evidence of the cumulative effect of ACEs and the mitigating role of social support on depressive symptoms draws attention to the need for intervention with young people living in contexts where adversities accumulate and overlap, causing fear, sadness, and threat. Furthermore, we highlight the need of previous interventions, as many of these adverse situations are avoidable and can be prevented. The protective action of schools and healthcare services is of paramount importance, which must be attentive to critical life situations, such as violence and poverty — which, in turn, cause great suffering.

Thus, future recommendations are directed to the development of early intervention programs with identification of signs of emotional distress in children and adolescents, strengthening of social support, training of teachers and healthcare professionals to recognize signs of stress and depression, promotion of safe environments, integration of services (schools, mental health services, and social organizations), and interventions focused on specific contexts.
