# Supplementary material for: Depressive symptoms of school-aged adolescents: cumulative effect of adverse events and mediation of social support
Source: Rev Bras Epidemiol. 2025 May 9;28:e250019. doi: 10.1590/1980-549720250019 (PMC12068810; doi:10.1590/1980-549720250019)
Supplement: Translation

# Translation: Sintomas depressivos de adolescentes escolares: efeito cumulativo de eventos adversos e mediação do apoio social

**DOI:** 10.1590/1980-549720250019.2

**Authors:** Célia Regina de Andrade, Raquel de Vasconcellos Carvalhaes de Oliveira, Joviana Quintes Avanci

**Affiliations:** I Fundação Oswaldo Cruz, Escola Nacional de Saúde Pública Sergio Arouca, Programa de Pós-Graduação em Saúde Pública, Departamento de Métodos Quantitativos em Saúde – Rio de Janeiro (RJ), Brasil.; II Fundação Oswaldo Cruz, Instituto Nacional de Infectologia Evandro Chagas. Programa de Pós-Graduação em Epidemiologia em Saúde Pública – Rio de Janeiro (RJ), Brasil.; III Fundação Oswaldo Cruz, Escola Nacional de Saúde Pública Sergio Arouca, Programa de Pós-Graduação em Saúde Pública, Departamento de Estudos sobre Violência e Saúde Jorge Careli – Rio de Janeiro (RJ), Brasil.

**Keywords:** Adolescência, Depressão, Apoio social, Experiência adversas na infância

## Abstract

**Objetivo:**

Analisar o efeito do acúmulo de experiências adversas na infância (EAI) e sintomas depressivos na adolescência, bem como identificar relações diretas e indiretas entre as variáveis e o papel mediador do apoio social.

**Métodos:**

Estudo transversal com 1.117 adolescentes escolares de 13 a 19 anos, usando o Inventário de Depressão Infantil e escalas EAI organizadas nas dimensões socioeconômica, familiar, comunitária e total. Realizaram-se análise descritiva e modelo de equação estrutural com estimativa de probit, aplicando o apoio social como mediador.

**Resultados:**

A prevalência de sintomas depressivos entre adolescentes escolares foi de 8,2%. Observou-se que o acúmulo de fatores socioeconômicos, familiares e comunitários aumenta a ocorrência de sintomas depressivos, ajustado por sexo, cor da pele e apoio social. A mediação pelo apoio social explica 16,7% do efeito total da exposição aos fatores analisados. Na dimensão socioeconômica, o probit de depressão aumenta 0,033 a cada categoria, com 21,2% mediado pelo apoio social. Na dimensão familiar, o aumento é de 0,020 (25% mediado), na comunitária de 0,018 (16,7% mediado) e na dimensão total de 0,012 (17% mediado).

**Conclusão:**

A constatação do efeito do acúmulo de EAI nos sintomas depressivos na adolescência requer ações em grupos vulnerabilizados, com o apoio social ajudando a mitigar o risco de depressão.

## INTRODUÇÃO

A depressão tem se tornado relativamente comum em todo o mundo e, na última década, aumentou em quase 20%, com estimativa de 322 milhões de indivíduos ou 4,4% da população mundial com quadro depressivo^1^. O Brasil ocupa a segunda posição de maior número de pessoas com depressão nas Américas, ficando em primeiro lugar na comparação entre países latino-americanos^1^.

Em todo o mundo, adolescentes são grupo de risco para depressão, com estimativa de 6,2 a 25% para transtorno depressivo maior^2^. O risco cumulativo de ocorrência de um ou mais episódios depressivos aumenta de 5 a 20% nesta fase da vida^3^. Na adolescência, a depressão é associada com dificuldades escolares, sociais, precária saúde física, suicídio e recorrência de episódios depressivos na vida adulta^2,4^. Os fatores de risco que se destacam são o histórico familiar de depressão e a exposição às experiências adversas na infância (EAI)^5^.

As EAI referem-se a traumas ou experiências negativas ocorridas até os 18 anos, com potencial de prejudicar a saúde e o bem-estar em longo prazo^6^. Desemprego, violência, doenças, perdas e ambientes familiares disfuncionais e instabilidade da estrutura familiar são adversidades estudadas que podem acentuar a depressão em adolescentes^7^. Adicionalmente, violência comunitária, discriminação racial e pobreza também são consideradas EAI^7^.

No Brasil, a situação de crianças, adolescentes e suas famílias piorou nos últimos anos. Exploração sexual, abandono, perdas, fome e violências afetam significativamente esse grupo. Entre 2016 e 2020, 35 mil jovens de 0 a 19 anos foram mortos de forma violenta. Além disso, de 2017 a 2020, 180 mil sofreram violência sexual — média anual de 45 mil por ano^8^. O aumento do desemprego desde 2015, especialmente entre mulheres (16,8%), impacta a vida e a saúde desses jovens^8^.

A perspectiva do risco ecológico cumulativo estuda fontes de risco provenientes de diversos contextos de vida, como família, escola, comunidade e pares, que operam concomitantemente ou em sequência^9^. Essa teoria, baseada em resposta ao estresse, sugere que exposições acumuladas afetam os sistemas reguladores fisiológicos em longo prazo^10^. Isso pode prever psicopatologia persistente da infância à idade adulta^11^ e transtornos psiquiátricos na adolescência^12^. O estresse gerado pelas EAI prediz 21% dos sintomas de depressão em adolescentes^13,14^.

No entanto, ainda se sabe pouco sobre os mecanismos mediadores e moderadores subjacentes à relação entre depressão e risco cumulativo de EAI, o que poderia indicar potenciais fatores de proteção para prevenção da depressão. Esses fatores podem modificar ou melhorar a resposta a um evento adverso e impedir o desenvolvimento do quadro depressivo^15^.

O modelo ecológico-transacional da psicopatologia do desenvolvimento organiza os fatores de proteção em vários níveis:

1. O individual, como as habilidades de enfrentamento positivas, de autorregulação, *locus* interno de controle, espiritualidade e alta autoestima,
2. O familiar, entre os quais os relacionamentos de apoio, e
3. O comunitário mais amplo, como por exemplo a conexão com atividades ou grupos culturais, a alta coesão social de um bairro e o apoio comunitário^15^.

O apoio social é um fator protetor estudado em modelos teóricos sobre depressão. O modelo de causalidade social sugere que o suporte social, ao proporcionar experiências positivas de afeto e cuidado, atenua a probabilidade de depressão, fortalecendo a autoestima e reduzindo a cognição negativa^16^. Níveis reduzidos de apoio percebido preveem sintomas depressivos na adolescência^17^. Além disso, adolescentes que percebem que podem contar com pessoas de confiança são capazes de reduzir os efeitos negativos da depressão ocasionados pelos eventos estressantes da vida^17^.

É estratégico compreender o papel de potenciais adversidades nos desfechos de saúde mental, assim como refletir sobre o efeito do acúmulo de experiências adversas e a ocorrência da depressão em adolescentes^18^. É ainda mais estratégico conhecer o papel mediador dos fatores de proteção, como o apoio social diante da exposição a situações adversas, a fim de orientar ações de intervenção direcionadas para crianças e adolescentes expostos a diferentes formas de EAI^19^. Este artigo busca analisar o efeito do acúmulo de experiências adversas na infância (EAI) e sintomas depressivos na adolescência, bem como identificar relações diretas e indiretas entre as variáveis e o papel mediador do apoio social.

## MÉTODOS

### Desenho e população do estudo

O estudo tem delineamento transversal com adolescentes de escolas públicas e privadas no município de São Gonçalo e foi realizado no ano de 2011. Este estudo faz parte de um projeto maior desenvolvido pelo Departamento de Estudos sobre Violência e Saúde Jorge Careli da Fiocruz.

O município de São Gonçalo é o segundo mais populoso do Estado do Rio de Janeiro^20^. Na ocasião da pesquisa, tinha população estimada em 999.728 hab.^20^, das quais um terço são crianças e adolescentes 328.829 de 0 a 19 anos, e população no último censo (2022) de 896.744 habitantes^20^. O Índice de Desenvolvimento Humano (IDHM) era de 0,739 em 2010, ocupando o 14° lugar entre as cidades do Rio de Janeiro. A faixa etária da adolescência representa cerca de 16% da população do município e, em 2022, a taxa de mortalidade infantil era de 12,56 por mil nascidos vivos^20^. No que se refere à violência contra a criança e o adolescente, em 2017, o Disque 100, canal da Ouvidoria Nacional dos Direitos Humanos, registrou mais de 84 mil denúncias de violação dos direitos de crianças e adolescentes, sendo mais de 20% relacionadas à violência sexual^21^.

Apesar dos altos índices de violência infantil no município, as políticas públicas voltadas para crianças e adolescentes vítimas ainda são incipientes. Destacam-se o Núcleo Especial de Atendimento a Crianças e Adolescentes Vítimas de Violência (NEACA), parte do fluxograma intersetorial, e o Programa Sentinela, que contribuem para a rede de atendimento e proteção desses jovens^20^.

A amostra incluiu adolescentes de 13 a 19 anos, estudantes do 9° ano de escolas públicas e privadas do município em 2011. Composta de 694 alunos de escolas públicas e 423 de particulares, foi dimensionada para estimativas de proporção de 2,3%, com erro de 1,3% e nível de confiança de 95%. Foram considerados 14 estratos conforme as áreas de planejamento e o tipo de escola. Utilizou-se uma amostragem conglomerada em multiestágio, com seleção aleatória de escolas e turmas. No total, 1.117 adolescentes de 43 escolas públicas e 30 particulares participaram, com cerca de duas turmas por escola.

A pesquisa foi aprovada pelo Comitê de Ética da Escola Nacional de Saúde Pública/Fundação Oswaldo Cruz (FIOCRUZ). A direção das escolas e os responsáveis assinaram o Termo de Consentimento, e os adolescentes, o Termo de Assentimento.

### Instrumentos de pesquisa

O questionário autopreenchível foi aplicado por turma, por equipes treinadas, sendo um dos membros psicólogo. O questionário foi composto de itens e escalas validadas previamente e utilizadas em estudos internacionais e com população brasileira, em que a variável de exposição utilizada são as experiências adversas na infância (EAI).

#### Experiências Adversas na Infância (EAI)

Trinta e três itens foram investigados com base em duas dimensões: eventos de vida traumáticos^22^ — estar em desabamento, desastre, incêndios, enchentes; acidente grave; conflito armado ou tiroteio; ele próprio/um familiar apanhar, levar um soco ou chute em casa; ser espancado, levar tiro ou ser ameaçado; testemunhar espancamento, ver alguém levar tiro ou ser morto; ver um cadáver em sua cidade; ter partes sexuais íntimas tocadas por adulto; ter recebido notícia de morte violenta ou ferimento grave de um ente querido; ter recebido um tratamento médico assustador e doloroso num hospital quando estava muito doente ou seriamente ferido; ter vivido outra situação assustadora, perigosa ou violenta; e escala de Trombeta e Guzzo^23^, com estes itens: desemprego de um dos pais/responsável; problemas financeiros sérios na família; falta comida em casa; mora ou já morou amontoado, sem espaço; problemas médicos sérios de familiares; problema físico ou mental na família; alguém na família indiciado ou preso; morte de pai ou mãe; parente próximo que morreu; problema de álcool ou droga na família; experiência sexual que envolveu os pais; discriminação pela cor de pele; separação de amigos por briga ou morte; ver alguém ser gravemente ferido; viver em perigo ou insegurança na vizinhança; ter a casa assaltada ou roubada.

#### Violência severa da mãe e/ou do pai contra o adolescente.

Foi utilizada a Escala Tática de Conflitos^24^, validada no Brasil^25^, composta de seis itens que variam de agressões físicas leves a graves. As respostas vão de "muitas vezes" a "nunca", e uma resposta positiva indica a presença de violência. Em estudo similar, o alpha de Cronbach para violência severa foi de 0,85 para mães e 0,92 para pais^26^.

#### Violência psicológica^27,28^

Caracteriza-se por 18 itens relativos a atos de humilhação, críticas excessivas e uso de palavras depreciativas contra o adolescente cometidos por pessoas significativas, com opções de respostas que variam do nunca até o sempre. Foi testada quanto à validade de constructo, com correlação positiva com sofrimento psíquico^29^, violência cometida pela mãe e entre pais^25^. O Kappa variou entre 0,395 e 0,683 e o alpha de Cronbach encontrado foi de 0,930^28,29^.

#### Violência na escola e na localidade

A Escala de Violência na Escola e na Comunidade, do Instituto Latino Americano das Nações Unidas para Prevenção do Delito e Tratamento do Delinquente (ILANUD)/Organização das Nações Unidas (ONU)^30^, avalia a vitimização de adolescentes na escola e comunidade no último ano. Um item positivo caracteriza o adolescente como vítima de violência no contexto correspondente. O coeficiente de Kuder-Richardson foi de 0,52 para violência escolar e 0,57 para violência comunitária, aceitável em razão do baixo número de itens^30^.

#### Violência entre os pais (ou padrasto/madrasta) e entre os irmãos

Avaliada por brigas entre si a ponto de se machucarem e humilharem. Uma ou mais respostas positivas em cada relação torna um caso na vitimização aferida^26^. Obtiveram-se bons coeficientes psicométricos na avaliação desses constructos: na violência entre os irmãos, o alfa de Cronbach obtido foi de 0,83, o índice de correlação intraclasse (ICC) foi de 0,6 e kappa moderado; para a que ocorre entre os pais, constatou-se ICC de 0,68^30,31^.

Os 28 itens das EAI e violências foram organizados e avaliados por:

1. Escore total, mensurado pelo somatório de todos os itens positivos; e
2. Escore por três dimensões — socioeconômico, familiar e da comunidade (Tabela 1).

**Tabela 1:** Prevalência das experiências adversas na infância segundo sintomas depressivos entre 1.117 adolescentes escolares de São Gonçalo, Rio de Janeiro, Brasil.

Experiências adversas		Sintomas Presença Prev% (n=92)	Depressivos Ausência Prev% (n=1025)	p-valor*
Dimensão socioeconômica				
	Seus pais ou responsável está desempregado	67,2	58,0	**0,061**
	Problemas financeiros sérios	47,4	37,1	**0,057**
	Falta comida em casa	5,6	2,1	**0,081**
	Mora ou morou amontoado	12,5	6,7	**0,071**
Dimensão familiar				
	Apanhar, levar um soco ou chute muito forte em casa	37,3	18,3	**<0,001**
	Um adulto tocou suas partes sexuais íntimas contra a sua vontade	17,0	7,7	**0,008**
	Familiar indiciado ou preso	34,6	20,7	**0,007**
	Ver membro da família apanhar, levar um soco ou chute muito forte em casa	39,6	22,6	**<0,001**
	Violência psicológica	55,3	14,4	**<0,001**
	Ter recebido notícia de morte violenta de um ente querido	38,9	41,7	0,656
	Problemas médicos com familiares	66,7	69,5	0,586
	Problema de deficiência física ou mental com familiar	33.5	24,0	**0,071**
	Morte de pai ou mãe	6,5	6,8	**0,928**
	Parente próximo que morreu	75,1	72,4	0,625
	Problemas de álcool ou drogas com familiares	41,6	31,2	**0,059**
Dimensão comunitária				
	Ser espancado, levar tiro ou ser ameaçado	30,2	10,8	**<0,001**
	Violência na escola	61,9	38,6	**<0,001**
	Violência na localidade	64,6	35,9	**<0,001**
	Separou de amigos por briga ou morte	62,8	30,9	**<0,001**
	Viu alguém ser gravemente ferido	48,2	37,0	**0,045**
	Situação de perigo e insegurança na vizinhança	33,1	25,5	0,129
	Casa assaltada ou roubada	12,3	11,6	0,825
	Desastres/incêndios/enchentes	21,0	12,1	**0,049**
	Estar em um acidente grave	19,3	11,3	**0,015**
	Estar em conflitos armados (tiroteios)	39,6	35,9	0,484
	Ver alguém na sua cidade ser espancado, levar um tiro ou ser morto	47,2	34,2	**0,053**
	Ver um cadáver em sua cidade	56,7	49,9	0,390
	Desabamento que destruiu o local em que estava	10,8	5,4	**0,058**

* Valores em negrito representam p≤0,10, testes estatisticamente significativos.

As opções de respostas ordinais da dimensão socioeconômica foram de 0–2 e mais; da dimensão familiar, de 0 a 2–5 e mais; da dimensão comunitária, de 0 a 2–5 e mais; e da dimensão total, 0 a 7–8 e mais (Tabela 2).

**Tabela 2:** Modelagem do efeito (total, direto e indireto) do escore das dimensões de experiências adversas na infância em relação aos sintomas depressivos, mediado pelo apoio social entre escolares adolescentes (n=1.117).

Dimensão		Efeito total (c’+ab)	Efeito direto (c’)	Efeito indireto (a*b)	Proporção da mediação†%
Socioeconômica‡					
	0	-	0,026	0,007	21,2
	1	0,033			
	2 e mais	0,066			
Familiar§					
	0 a 2	-	0,015	0,005	25,0
	3 a 4	0,020			
	5 e mais	0,040			
Comunitária//					
	0 a 2	-	0,016	0,003	16,7
	3 a 4	0,018			
	5 e mais	0,036			
Total^g^					
	0 a 7	-	0,010	0,002	16,7
	8 e mais	0,012			

* Os efeitos foram ajustados por sexo e raça/cor da pele autorreferida;

† Proporção da Mediação=efeito indireto (a*b)/efeito total(c’+ab);

‡ Modelo Socioeconômico CFI=0,972, RMSEA=0,048;

§ Modelo Familiar CFI=0,971, RMSEA=0,058;

// Modelo Comunitário CFI=0,987, RMSEA=0,037;

¶ Modelo Total CFI=0,986, RMSEA=0,043.

#### Sintomas depressivos

O Inventário de Depressão Infantil (CDI), desenvolvido por Kovacs^32^, avalia a presença e a severidade dos sintomas depressivos em crianças e adolescentes, adaptado do Inventário de Depressão de Beck (BDI) para adultos. No Brasil, foi traduzido e adaptado por Gouveia et al.^33^, mantendo os 27 itens da versão original. O CDI permite o rastreamento de sintomas depressivos em indivíduos de 7 a 17 anos, abordando sintomas afetivos, cognitivos, somáticos e comportamentais^34,35^ nas últimas duas semanas. A escala de resposta tem três opções: ausência, presença e gravidade^35^. Suas propriedades psicométricas são bem estabelecidas, com coeficientes alfa variando entre 0,18 e 0,55^36,37^. O escore é obtido pela soma dos itens, com valores acima de 65 indicando possíveis casos de depressão^37^.

#### Apoio social

A versão reduzida da subescala de apoio social do MOSSSS, com seis itens e um fator^38^, foi adaptada e validada para o Brasil^39^, apresentando boas medidas de confiabilidade e validade^40,41^. Essa versão avalia o suporte social funcional global (apoio material, afetivo, interação social positiva e apoio emocional/informacional) e mostra alta consistência interna. A média é utilizada como ponto de corte para classificar o apoio social em duas categorias: de 7 a 25 pontos, ausência de apoio social; de 25 a 30, presença de apoio social^39^.

As variáveis sexo (masculino e feminino) e cor da pele autorreferida (branca, preta/parda e amarela/indígena) foram estudadas como covariáveis sociodemográficas.

### Análise dos dados

Os itens do questionário foram codificados e digitados em uma máscara de dados no Epidata 3.1. Realizou-se análise descritiva para obter a prevalência de sintomas depressivos e outras variáveis. Em seguida, associaram-se os itens das EAI e violências com a variável de sintomas depressivos (presente/ausente), comparando as proporções pelo teste Wald, com nível de significância de 10%.

As variáveis significativas ao nível de 10% foram selecionadas para construir as quatro variáveis de exposição relacionadas às EAI: três dimensões (socioeconômico, familiar e comunitário) e o escore total. Os oito eventos de EAI (como morte de ente querido, problemas médicos com familiares e conflitos armados) foram incluídos nas dimensões por sua relevância teórica. Cada variável de exposição foi criada somando-se os eventos (1 se presente, 0 se ausente) e, em seguida, categorizada em duas ou três categorias conforme a quantidade de eventos.

Posteriormente, foi aplicado o modelo de equações estruturais para desfecho binário (com função de ligação probit) para avaliar os efeitos de exposição de cada uma das três dimensões e do escore total de EAI no desfecho de sintomas depressivos. As variáveis de exposição (EAI) foram tratadas como variáveis ordinais na estimação (Tabela 2).

Os efeitos foram interpretados a cada mudança na categoria das dimensões de EAI e os modelos foram ajustados pelas variáveis sexo (masculino e feminino) e raça/cor da pele autorreferida (brancos e pretos — preto/parda/amarela/indígena), além de considerar a variável apoio social como variável mediadora (Figura 1). Foi considerado o nível de significância de 5% nos testes dos coeficientes do modelo.

**Figura 1:** Diagrama do efeito (total, direto e indireto) do escore das dimensões de experiências adversas na infância nos sintomas depressivos mediados pelo apoio social.

NOTA: a=efeito da exposição - >mediadora; b=efeito de mediadora; c=efeito total; c’=efeito direto ou *average direct effects* (ADE)

a*b=efeito indireto ou *average causal mediation effects* (ACME), c=c’ + a*b=*average direct effects* (ADE) *average causal mediation effects (ACME).

EAI: experiências adversas na infância.

O efeito total de cada dimensão e total do EAI no desfecho de sintomas depressivos foi composto de um efeito direto e um indireto, ou seja, mediado por apoio social (Figura 1). O objetivo da análise de mediação foi estimar uma partição do efeito total em um efeito indireto da exposição no desfecho via mediação e um efeito direto da exposição no desfecho^42,43^. Se existir um efeito de EAI via mediação (efeito indireto), será observado que a proporção entre o efeito indireto e total será razoável. Consideramos valores acima de 10% de proporção de mediação como um potencial efeito.

Para avaliar a qualidade de ajuste do modelo, foram utilizadas as medidas *comparative fit index* (CFI) e *root mean square error of aproximation* (RMSEA), considerando-se um ajuste adequado quando CFI acima de 0,9 e RMSEA abaixo de 0,05^44^.

As análises foram feitas utilizando a biblioteca *survey* ^45^ do pacote estatístico R e incorporando o peso e plano amostral em todas as análises. Para a modelagem estrutural foi utilizado o pacote *lavaan.survey* ^46^ (versão 06-19) do *software* R, versão 4.4.1 (http://www.r-project.org)^45^.

## RESULTADOS

Dos 1.117 participantes, a prevalência de sintomas depressivos foi de 8,2% (intervalo de confiança de 95% — IC95% 6,7–9,9). Dos 33 eventos adversos e violências estudadas, 20 apresentaram diferenças estatísticas significativas na presença ou ausência de sintomas de depressão. Na dimensão socioeconômica, destacam-se o desemprego dos pais, sérios problemas financeiros, ausência de comida e morar amontoado. Na dimensão da família, destacam-se a vitimização ou o testemunho da violência física, violência sexual, violência psicológica, prisão de familiar, deficiência física ou mental e problemas com álcool ou drogas. Na dimensão da comunidade, ressaltam-se a violência da comunidade, da escola, a separação de amigos por briga ou morte, o testemunho de violência na cidade, acidente, desastres/incêndios/enchentes e desabamento (Tabela 1).

As dimensões de EAI foram construídas pela soma de eventos dos 20 itens significativos na Tabela 1, mais oito selecionados pela relevância teórica e, posteriormente, categorizados. A Tabela 2 mostra os resultados do modelo de equações estruturais aplicado a cada dimensão e ao escore total de EAI, ajustado por raça/cor da pele autorreferida e sexo, considerando-se a mediação por apoio social. Observa-se que a análise das EAI apresenta um efeito gradiente, pois foi cumulativo de acordo com as categorias de cada dimensão. Na dimensão socioeconômica, a cada aumento de uma categoria se aumenta em 0,033 o probit de ocorrência de depressão, quando ajustado por sexo e cor da pele, sendo, todavia, 21,2% desse efeito total mediado pelo apoio social. Com relação à dimensão familiar, o aumento se dá em 0,020 e 25,0% é mediado pelo apoio social; na dimensão comunitária, o aumento é de 0,018 e 16,7% mediado pelo apoio social. Por fim, na dimensão total, o aumento é de 0,012 e 16,7% mediado pelo apoio social. As medidas de qualidade de ajuste dos quatro modelos encontraram-se em conformidade com os parâmetros esperados (Tabela 2).

## DISCUSSÃO

O estudo traz relevante contribuição para o entendimento da relação complexa entre adversidades e depressão. Primeiramente, a prevalência de 8,2% de sintomas depressivos entre adolescentes escolares dialoga com pesquisas em saúde mental na faixa etária, que encontram percentuais entre 7 e 10%^47,48^.

Todavia, dois principais achados se destacam para o desfecho da depressão na adolescência:

1. O gradiente de efeito diante do acúmulo de eventos adversos e
2. O papel mediador do apoio social, que atenua a ocorrência da depressão, mesmo que se não opere de forma a impedir a sua ocorrência.

A relação dose-resposta entre o aumento das experiências adversas na infância e a maior ocorrência de sintomas depressivos em adolescentes destaca o impacto negativo cumulativo dessas experiências^49^. Cada novo evento adverso em cada dimensão estudada aumenta progressivamente a ocorrência de sintomas depressivos. Esse achado é relevante, pois aponta para adolescentes que, por suas condições de vida ou saúde mental, vivem em contextos que se retroalimentam por experiências adversas, podendo desencadear/agravar seu quadro atual ou futuro, incluindo transtornos alimentares e de personalidade^50-52^. A situação é preocupante, pois muitas dessas experiências adversas são mantidas em segredo e não são reconhecidas ou valorizadas pelas pessoas ao redor do jovem^13^.

Quanto ao apoio social, sabe-se do seu papel de proteção para o desenvolvimento da depressão e de outros transtornos mentais^50^, tendendo a mitigar os impactos nefastos das adversidades^18,51^. Adolescentes que recebem ajuda, apoio e são ouvidos podem ver situações difíceis como menos ameaçadoras e desenvolver atitudes mais positivas. No entanto, o estudo mostra que o apoio social sozinho não impede o impacto cumulativo das experiências adversas na infância (EAI) no desenvolvimento de sintomas depressivos. Experiências de pobreza, violências, acidentes, perdas e doenças na infância colocam os adolescentes em risco de problemas de saúde mental, influenciando etapas posteriores da vida^52^. A relação mediadora do apoio social independe do sexo ou raça do adolescente, o que requer cautela e mais estudos futuros.

Nos resultados encontrados entre as dimensões, os maiores efeitos na depressão foram na dimensão socioeconômica, seguida da familiar. O apoio social tem um efeito mediador maior também nas duas dimensões citadas.

Recomenda-se estudar os achados com base no Modelo Dimensional das Adversidades e Psicopatologia^53^, que diferencia eventos de ameaça (como violência física e sexual) e privação (como negligência). Esse modelo sugere que ameaças resultam em regulação de estímulos de medo, enquanto a experiência das privações prejudica a maturação cognitiva e a aprendizagem. Diferentes adversidades podem explicar a resposta interativa das EAI na depressão^54^. A violência está ligada ao processamento de ameaças e a depressão a déficits associados à perda^55^, demonstrando que os efeitos das adversidades podem ser independentes e mais bem compreendidos pela sua natureza das adversidades^56^.

A principal limitação deste estudo refere-se ao uso de questionário de autorrelato. Esse tipo de abordagem restringe informações sobre o contexto dos eventos e da depressão, além de ser sujeito a vieses de memória. Além disso, o humor atual do adolescente pode levar a excessos de relatos de adversidades e sofrimentos. Outro ponto é que a análise de questões de saúde mental anterior à pandemia de COVID-19 pode apresentar um cenário mais positivo de fenômenos como a depressão, requerendo uma compreensão mais crítica baseada no contexto atual. Como recomendação para estudos futuros, sugere-se a utilização de estudos longitudinais e a aplicação de abordagem qualitativa, que podem refinar a compreensão de causalidade dos fatores familiares e sociais que desencadeiam a depressão na adolescência.

Como conclusão, a evidência do efeito cumulativo das EAI e do papel atenuador do apoio social nos sintomas depressivos chama a atenção à necessidade de intervenção com os jovens que vivem em contextos em que as adversidades se acumulam e se sobrepõem, acirrando o medo, a tristeza e a ameaça. Também, de uma intervenção anterior, já que muitas dessas situações adversas são evitáveis e podem ser prevenidas. É de grande relevância a atuação protetora de escolas e serviços de saúde, que precisam estar atentos em situações de vida críticas, como as violências e a pobreza, e que produzem muito sofrimento.

Assim, recomendações futuras direcionam-se para o desenvolvimento de programas de intervenção precoce com identificação de sinais de sofrimento emocional em crianças e adolescentes, fortalecimento de apoio social, capacitação de professores e profissionais de saúde para reconhecer sinais de estresse e depressão, promoção de ambientes seguros, integração de serviços (escolas, serviços de saúde mental e organizações sociais) e intervenções focadas em contextos específicos.
